# Dicer is required for neural stem cell multipotency and lineage progression during cerebral cortex development

**DOI:** 10.1186/1749-8104-8-14

**Published:** 2013-07-29

**Authors:** Nathalie Saurat, Therese Andersson, Navneet A Vasistha, Zoltán Molnár, Frederick J Livesey

**Affiliations:** 1 Gurdon Institute, University of Cambridge, Tennis Court Road, Cambridge CB2 1QN, UK; 2 Department of Biochemistry, University of Cambridge, Tennis Court Road, Cambridge CB2 1QN, UK; 3 Wellcome Trust – Medical Research Council Cambridge Stem Cell Institute, University of Cambridge, Tennis Court Road, Cambridge CB2 1QR; 4 Department of Physiology, Anatomy and Genetics, University of Oxford, Oxford, UK

**Keywords:** Cerebral cortex, Stem cells, Dicer, microRNA, Multipotency

## Abstract

**Background:**

During cerebral cortex development, multipotent neural progenitor cells generate a variety of neuronal subtypes in a fixed temporal order. How a single neural progenitor cell generates the diversity of cortical projection neurons in a temporal sequence is not well understood. Based on their function in developmental timing in other systems, Dicer and microRNAs are potential candidate regulators of cellular pathways that control lineage progression in neural systems.

**Results:**

Cortex-specific deletion of Dicer results in a marked reduction in the cellular complexity of the cortex, due to a pronounced narrowing in the range of neuronal types generated by Dicer-null cortical stem and progenitor cells. Instead of generating different classes of lamina-specific neurons in order over the 6-day period of neurogenesis, Dicer null cortical stem and progenitor cells continually produce one class of deep layer projection neuron. However, gliogenesis in the Dicer-null cerebral cortex was not delayed, despite the loss of multipotency and the failure of neuronal lineage progression.

**Conclusions:**

We conclude that Dicer is required for regulating cortical stem cell multipotency with respect to neuronal diversity, without affecting the larger scale switch from neurogenesis to gliogenesis. The differences in phenotypes reported from different timings of Dicer deletion indicate that the molecular pathways regulating developmental transitions are notably dosage sensitive.

## Background

Common features of neural stem cells in all regions of the nervous system are their multipotency and their ability to generate complex neuronal lineages in a fixed temporal order before switching to gliogenesis. In the cerebral cortex, glutamatergic projection neurons of each layer are generated by cortical stem and progenitor cells in a well-described order [1]. Subplate and deep, layer 6 neurons are generated first, followed by layer 5 projection neurons, including corticospinal motor neurons, before the production of upper layer neurons that populate layers 2 to 4 [1]. The ability of cortical progenitor cells to produce projection neurons of different classes in a reproducible order is a cell autonomous property that is recapitulated by single cortical progenitor cells *in vitro* at clonal density [2-6]. However, the cellular and molecular mechanisms controlling cortical stem/progenitor cell multipotency and lineage progression are currently not well understood.

MicroRNAs have been considered as strong candidates for contributing to a cellular mechanism that controls multipotency and lineage progression in the nervous system [7]. Some *C. elegans* heterochronic genes, a key class of genes regulating developmental timing, encode microRNAs [8]. Individual microRNAs and microRNA families have been found to have a diverse set of functions in the developing nervous system, including neurogenesis (for review, see [9]). For example, mir-9 has roles in the regional specification of neural progenitor cells in the developing vertebrate central nervous system [10], and mir-124, one of the most abundant neuronal microRNAs, regulates neurogenesis and neuronal differentiation [11,12].

Previous studies have found that Dicer, the RNase critical for the generation of most miRNAs, is necessary for differentiation but not self-renewal of neural stem cells [13,14]. Previous analyses of phenotypic outcomes following deletion of Dicer in the developing nervous system reported a range of phenotypes of differing severity, and often contradictory outcomes. This appears to depend, in part, on the timing of the removal of Dicer function: deletion before the onset of neurogenesis results in greatly increased cell death and an overall reduction in neuronal number [15,16], whereas deletion slightly later leads to a milder phenotype with less cell death and a reduction in upper layer neurons [16]. Knockout of Dicer in the early developing forebrain using a FoxG1-Cre knock-in line demonstrated the requirement for Dicer in the production of neurogenic radial glial cells from neuroepithelial cells, but found that the relative proportions of different cortical neuronal types appeared normal [17]. In the developing retina, however, Dicer deletion early in development results in increased production of early born cell types and a reduction in the genesis of late born cell types [18]. Astrocyte differentiation was increased in the cortex of Dicer mutants generated using Nestin-Cre [19], which deletes slightly later than both FoxG1-Cre and Emx1-Cre. This is in contrast with Dicer deletion in the spinal cord, which found that Dicer is required for the switch from neurogenesis to gliogenesis [20]. Removal of Dicer from neurons in the hippocampus, retina and cerebellum results in neurodegeneration and neuronal loss [21-23].

To resolve some of the contradictions in the existing studies, and to address the potential role for Dicer and Dicer-regulated pathways in controlling developmental timing and neuronal lineage specification, we generated a cortex-specific Dicer mutant in which Dicer is deleted before the onset of neurogenesis. In contrast with previous reports, we find that Dicer loss of function leads to pronounced changes to the relative numbers of different classes of cortical neurons generated, such that excessive numbers of early born, deep layer neurons are produced throughout development, at the expense of upper layer neurons, which are almost completely absent. Moreover, absence of Dicer alters developmental timing, such that cortical stem/progenitor cells continuously produce the first cells in the lineage, demonstrating reduced multipotency and failure of lineage progression. In spite of the failure in neuronal lineage progression, the switch to gliogenesis still took place in late gestation. These data indicate that Dicer-regulated pathways have key roles in regulating multipotency, lineage progression and the timing of neurogenesis in the cerebral cortex without affecting the larger scale switch from neurogenesis to gliogenesis. We conclude that the variation in phenotypes reported following Dicer deletion at different stages of development may reflect the dosage sensitivity of many cellular pathways that regulate neural development.

## Methods

### Mice

Cortex-specific, Dicer conditional mutants were generated by crossing Emx1-Cre mice [24] with mice carrying floxed alleles of Dicer [25]. The plug date was taken as E0.5 and birthdate was designated postnatal day 0 (P0). Tissue was fixed in 4% PFA, cryopreserved and sectioned as described [26]. For birthdating, 40 mg/kg BrdU (B5002; Sigma-Aldrich) was injected intraperitoneally into timed pregnant dams at E14.5 and tissue collected at P0. All animal work was carried out in accordance with local and national (Home Office) ethical and legal regulations.

### miRNA microarray

miRNA expression profiling was carried out by Cambridge Genomic Services, Department of Pathology, University of Cambridge. Total RNA was extracted using Trizol (Sigma) from cortices from two individual E13.5 wild-type and two E13.5 Dicer null mice. Total RNA was hybridized to miRNA oligo arrays according to the manufacturer’s instructions (Toray). Expression levels were normalised against spiked control RNAs. Individual miRNA expression levels for the 40 most abundant miRNAs in the wild-type cortex were compared with expression in the Dicer null cortex, calculated as a percentage of wild type.

### Immunohistochemistry

Immunohistochemistry was performed on 12 μm sections as previously described [27]. The following antibodies were used in this study: rabbit anti-aCasp3 (9661L; Cell Signaling Technology), rat anti-BrdU (ab6326; Abcam), goat anti-Brn2 (sc-6029; Santa Cruz Biotechnology), rat anti-CTIP2 (ab18465; Abcam), rabbit anti-CDP (Cux1) (sc-13024; Santa Cruz Biotechnology), ER81 (gift from S. Arber), rabbit anti-FOXP2 (ab16046; Abcam), rabbit anti-GLUL (ab49873; Abcam), rabbit anti-GFAP (G9269; Sigma-Aldrich), rabbit anti-HMGA2 (ab41878; Abcam), mouse anti-Ki67 (550609, BD Biosciences), rabbit anti-Nurr1 (sc-990; Santa Cruz), rabbit anti-Pax6 (PRB-278P; Covance), rat anti-PH3 (ab10543; Abcam), mouse anti-Reelin (mab5364; Chemicon), rabbit anti-S100 (Z0311; Dako), mouse anti-Satb2 (ab51502; Abcam), rabbit anti-Tbr1 (ab31940; Abcam), rabbit anti-Tbr2 (ab2283; Millipore) and mouse anti-Tuj1 (MMS-435P; Covance). Sections were imaged on a Zeiss 510 Meta confocal microscope.

### Image analysis

Quantifications were performed on three sections per brain from two Dicer mutant and two wild-type embryos, with the exception of the cortical width analyses and the E15.5 aCasp3 and HMGA2 counts, for which three mice per genotype were used. For cell quantification, images were analysed using the NIH ImageJ ITCN plugin to count the total number of labelled nuclei. Volocity (Improvision) was used to count the number of Tbr1/BrdU double positive nuclei at P0. Student t-tests were used to identify significant differences in cell counts between the controls and the null mutants.

### RT-PCR

Total RNA was extracted from the P0 cortex using Trizol and 0.75 μg of total RNA was used to generate oligo-dT-primed cDNA using SuperScript III RT (Invitrogen). Quantitative PCR was carried out using SYBR Green JumpStart Taq ReadyMix (Sigma) on a 7300 Real-Time PCR System (Applied Biosystems) using 5 ng of cDNA per reaction and standard cycling conditions. The relative abundance of each gene was normalized to TBP and beta-actin and relative expression levels were calculated using the comparative Ct method [28].

Primer pairs used for RT-PCR and qRT-PCR: β-actin 5′- GTCGTCGACAACGGCTCCGGCATGTG- 3′, 5′- CATTGTAGAAGGTGTGGTGCCAGAT- 3′; Aldh1l1 5′- TTCATGGCAACAGAAGGTTG- 3′, 5′- GCTCGCAGCTAGCTAGCAGGTACT - 3′; GFAP 5′ -TTTCTCGGATCTGGAGGTTG- 3′, 5′ -AGATCGCCACCTACAGGAAA - 3′; Glul 5′ -TTGCTTGATGCCTTTGTTCA - 3′, 5′ -CTCCTGACCTGTTCACCCAT - 3′; Ntsr2 5′ -GGTACCTGGGAGTAGAGGGC - 3′, 5′ -CTAGTAAGTCGCGCCAGCTC - 3′; S100β 5′ -CCGGAGTACTGGTGGAAGAC - 3′, 5′ -GGACACTGAAGCCAGAGAGG - 3′; TBP 5′-ACTCCTGCCACACCAGCT-3′, 5′- AAGTGCAATGGTCTTTAGGTC -3′; βIII-tubulin 5′-CTGGAGCGCATCAGCGTAT-3′, 5′- GGTTCCAGGTTCCAAGTCCA-3′.

### Western blot

Imunoblotting was performed as previously described [27]. Briefly: total protein was extracted from wild-type and Dicer null E13.5 and E15.5 cortex (*n* = 2) using RIPA lysis buffer in the presence of 1× Complete protease inhibitor (11697498001; Roche). A total of 10–20 μg protein was loaded onto a 4-20% Tris-Glycine gel (Invitrogen) and transferred to PVDF (GE healthcare) membranes. Antibodies used for western blotting were: mouse anti- β-actin (A2228; Sigma-Aldrich) and mouse anti-Ezh2(AC22) (3147; Cell Signaling Technology). Band intensity was measured using NIH ImageJ and normalised to β-actin.

### DiI

For axonal tracing, single crystals of DiI (1, 1- dioctadecyl -3, 3, 3′,3′-tetramethyl-indocarbocyanine perchlorate; Molecular Probes), were placed with a fine tungsten wire into the dorsal thalamus, cerebral peduncle or dorsal cortex under a dissecting microscope [29]. Brains were incubated in 4% paraformaldehyde at room temperature (RT) for 3 weeks. The brains were then embedded in 4% agarose and 70 μm coronal Vibroslicer sections were cut. Tissue was counterstained with 0.1% DAPI. Sections were coverslipped in PBS and analysed with a fluorescent microscope (Leica DMR) or a laser scanning confocal microscope (Carl Zeiss LSM 710).

## Results

### Limited diversity of cortical projection neurons in the Dicer null cortex

**Figure 1:**
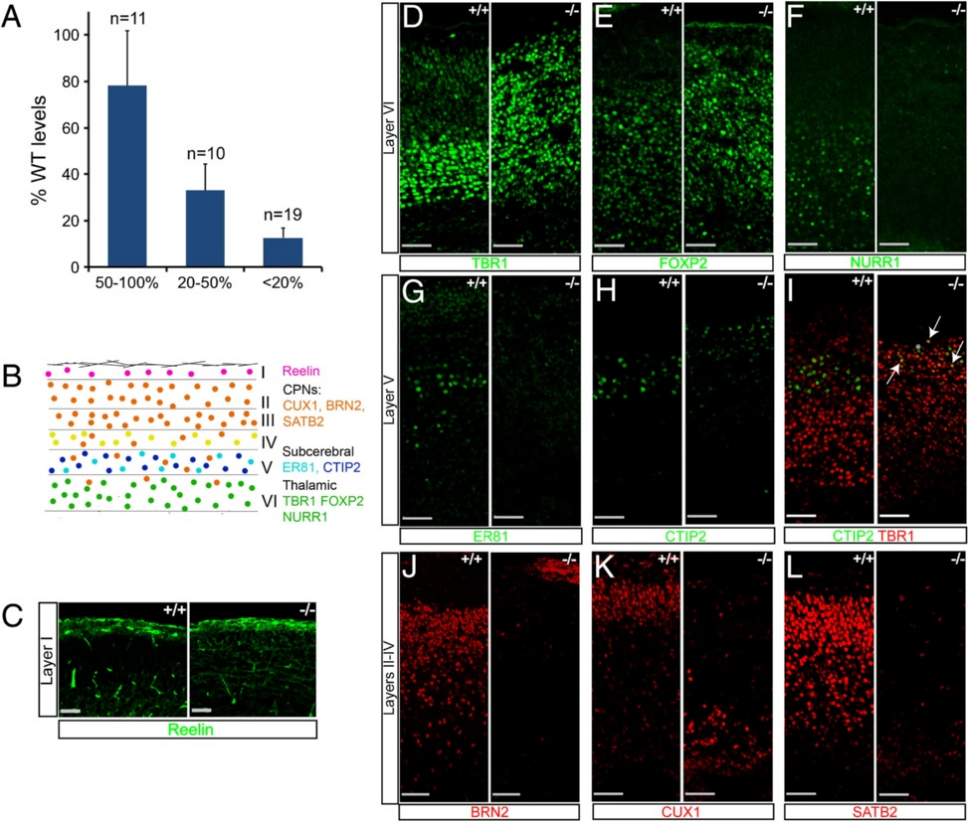
**Cortex specific knockout of Dicer results in an increase in Tbr1+ neurons and a near absence of other neuronal classes. (A)** Microarray analysis of the expression in the E13.5 Dicer null cortex of the 40 most abundant miRNAs in the wild-type E13.5 cortex identifies three groups of miRNAs, based on relative change in expression. Approximately half of the miRNAs are reduced to <20% of wild-type levels, but one-quarter show minor changes in levels. **(B)** Overview of the different classes of projection neurons, the cortical layers they occupy and the transcription factors used to identify them. **(C-L)** Representative immunofluorescence images for transcription factors that are differentially expressed in laminar/projection neuron types in the neonatal (P0) cortex. Immunostaining was performed on matched sections from three Dicer null and three control littermate embryos. **(D)** Progenitor cells lacking Dicer maintain the ability to generate Reelin+, layer 1 neurons. **(D-F)** The Dicer null cortex contains much larger numbers of layer 6 Tbr1+ **(D)** and FoxP2 neurons **(E)**, but lacks Nurr1+ subplate/layer 6A neurons **(F)**. **(F-H)** There was a severe reduction in layer 5, subcerebral projection neurons expressing ER81 **(G)** or CTIP2 **(H)**. Notably almost all of the few remaining CTIP2 expressing neurons also co-expressed Tbr1 (arrows), with very few CTIP2+/Tbr1^-^ cells present (asterisk) in the Dicer null cortex **(I)**. Layers 2 to 4 callosal projection neurons expressing Brn2 **(J)**, Cux1 **(K)** or Satb2 **(L)** were markedly decreased in the Dicer null cortex. CPNs, callosal projection neurons. Scale bars: 50 μm.

To generate cortex-specific loss of Dicer function, the cortex-specific Emx1-Cre driver line [24] was used in combination with a mouse strain carrying alleles of Dicer with loxP sites flanking an exon encoding one of the RNAse domains [25]. Analysis of the 40 most abundant miRNAs present in the wild-type cortex at E13.5, comparing the amounts of mature miRNAs in control and Dicer null cortices, found that miRNAs could be divided into three broad classes, based on their relative reduction in abundance (Figure 1A; Additional file 1): miRNAs that are reduced to <20% in abundance (19 of the 40 miRNAs sampled); miRNAs reduced by between 20% and 50% (10/40 miRNAs); and miRNAs reduced by between 50% and 100%.

Following cortex-specific loss of Dicer function, we noted a marked reduction in the size of the Dicer null cortex in mid-gestation and at birth. Analysis of the diversity of projection neuron types in the cerebral cortex at birth (P0) demonstrated a marked difference in cellular composition in the Dicer null cortex (Figure 1). In contrast to the discrete band of Tbr1-expressing subplate and layer 6 projection neurons found in the wild-type cortex, the Dicer null cortex contained large populations of Tbr1+ and Foxp2+ neurons that spanned the entire radial width of the cortex (Figure 1). No Nurr1-expressing subplate or layer 6 neurons were present in the Dicer null cortex, compared with the defined population present in wild-type control cortex (Figure 1). Furthermore, Er81-expressing, layer 5 neurons cells were almost completely absent in Dicer null cortices, and very few CTIP2+/Tbr1- layer 5 corticospinal motor neurons were present (Figure 1). Together, these data indicate that there is marked reduction in the complexity of infragranular, deep layer projection neurons in the Dicer null cortex.

Accompanying the changes in the number and composition of deep layer, infragranular neuron populations in the Dicer null cortex at birth, there was also a near absence of upper layer neurons. The Dicer null cortex was almost completely devoid of Brn2+ neurons (layer 2/3; Figure 1), and very few Cux1+ or Satb2+ layer 2 to 4 callosal projection neurons were found (Figure 1). The small numbers of Cux1+ neurons in the neonatal Dicer null cortex were clustered deep in the cortex, near the ventricular surface, rather than being located proximal to the pial surface, as seen in the wild-type cortex (Figure 1). However, Reelin + neurons were clearly present in layer 1 of the Dicer null cortex, excluding lack of Reelin as a contributory factor for the altered migration of the small number of upper layer neurons in the Dicer null cortex (Figure 1).

### Altered connectivity of the Dicer null cortex

**Figure 2:**
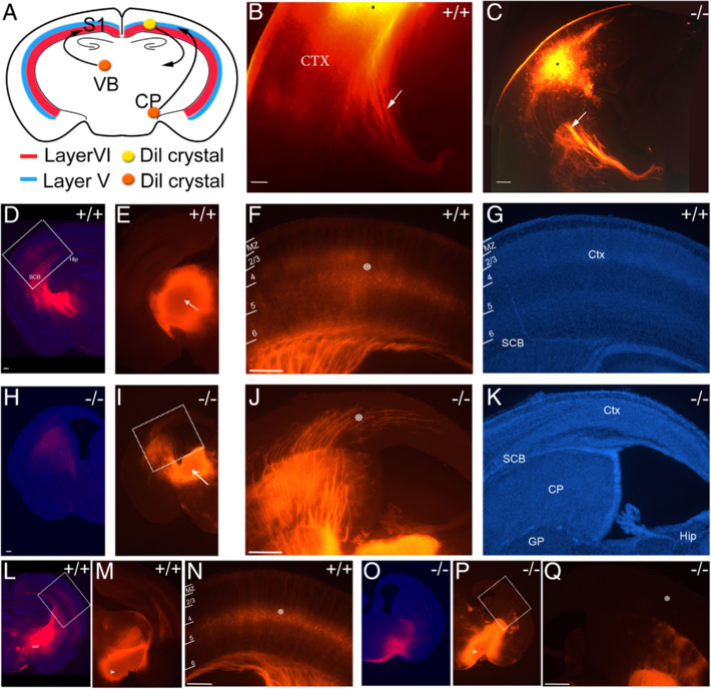
**Thalamocortical innervation of the P7.5 Dicer null cortex. (A)** Schematic diagram indicating DiI crystal placement in the ventrobasal thalamus and cerebral peduncle for tracing of corticothalamic and corticopeduncular projections (orange circles) and in the somatosensory cortex (yellow circle) to reveal corticofugal, corticopetal and callosal projections. **(B-C)** Crystal placement in the cortex (denoted by black asterisk) revealed thalamocortical connections that descend ventrolaterally to the VB. The axons change their fasciculation at the striato-cortical junction (see arrow in **C**) and extend in thicker fascicles through the striatum (arrow in **B**). **(D, E)** and **(H, I)** DiI crystal placement (see arrow) in the ventrobasal thalamus of the wild-type **(D, E)** and Dicer null **(H, I)** cortex. **(F, G)** Magnification of boxed area in **(E)** showing retrogradely labelled layer 5 and some layer 6 cortical projection neurons in the wild-type. **(J, K)** Magnification of boxed area in **(I)** showing the presence of deep layer specific thalamocortical projections that maintain some degree of fasciculation as they progress within the cortical plate. **(K)** DAPI stained image showing the reduced thickness of the cortical plate, loss of lamination and hugely reduced hippocampus in the Dicer null cortex. **(L, M, O-P)** DiI crystal placement (see arrow) in the cerebral peduncle of the wild-type **(L, M)** and Dicer null **(O, P)** cortex. **(N, Q)** Magnification of boxed areas in **(L)** and **(P)**, respectively, showing the retrograde labelling of layer 5 cortical neurons in the wildtype. **(Q)** There are no backlabelled cells apparent from similar cerebral peduncle crystal placements in the Dicer null, suggesting reduced corticofugal projections. White star depicts the cortical plate. Four brains from each genotype were analysed. CP, cerebral peduncle; CPu; caudate putamen, CTX, cortex; GP, globus pallidus; Hip, Hippocampus; S1, somatosensory nucleus; SCB, striatocortical boundary; VB, ventrobasal thalamus. Scale bars: 100 μm.

The changes in cellular composition in the Dicer null cortex reported above suggest that the Dicer null cortex contains large numbers of layer 6/subplate corticothalamic neurons, with few layer 5 corticospinal projection neurons. Tbr1+ neurons are positioned throughout the Dicer null cortical plate, which shows an overall reduction in thickness. Consistent with that observation, anterograde axonal tracing from the primary motor and somatosensory areas of the P7 Dicer null cortex, using lipophilic dyes, demonstrated the presence of a large corticothalamic projection (Figure 2B, C; *n* = 4 mice of each genotype). Furthermore, the anterograde-labelled cortical projections showed abnormal fasciculation patterns (Figure 2C).

Tracing from the ventrobasal thalamus (VB) and cerebral peduncle to identify thalamic and corticospinal projections confirmed these observations. Tracing from VB revealed labelling of cortical neurons in the wild-type and the Dicer null cortices, although axonal fasciculation and cell body placement in the cortical plate differed in the Dicer null cortex (Figure 2F and J). No projections to the cerebral peduncle were retrogradely traced to the Dicer null cortex, compared to the obvious labelling of the wild-type cortex, indicating an absence of layer 5 corticospinal neurons projecting subcortically to the cerebral peduncle (Figure 2O-Q). Finally, no contralateral projection or corpus callosum was present in the Dicer null cortex (data not shown), in agreement with the small numbers of Satb2+ upper layer projection neurons found at birth. Together, these data indicate that the Dicer null cortex contains layer 6 corticothalamic neurons that project appropriately to the thalamus, but lacks a corticospinal projection from layer 5 neurons.

### Increased numbers of deep layer neurons in the Dicer null cortex in later cortical development

**Figure 3:**
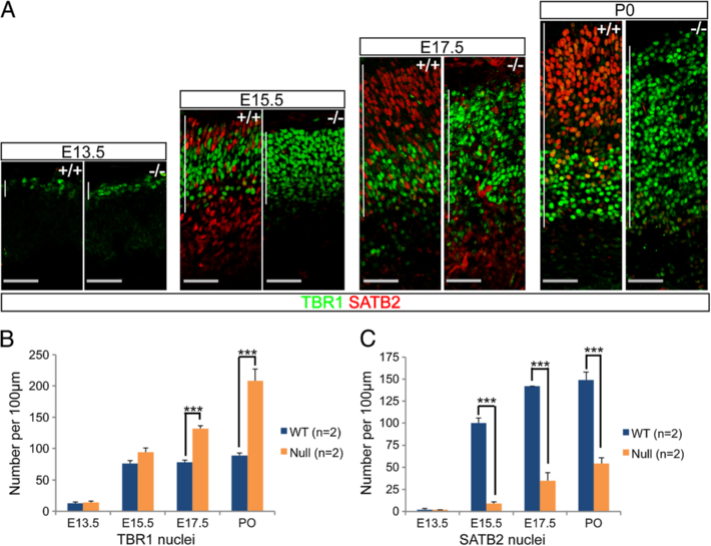
**The number of deep layer, Tbr1+ neurons continues to increase throughout the neurogenic period in the Dicer null cortex. (A)** Time course of Tbr1 (layer 6 transcription factor) and Satb2 (layer 2 to 4 transcription factor) expression in the developing cerebral cortex. In the wild-type the generation of deep layer neurons plateaus by E15.5 when the late born Satb2+ population begins expanding. Conversely, in the Dicer null cortex, the number of Tbr1+ cells increases throughout development and only a very limited number of upper layer neurons are produced during the neurogenic period. **(B, C)** Quantification of Tbr1 **(B)** and Satb2 **(C)** positive nuclei showed significant increases in Tbr1 nuclei number in the Dicer null at E17.5 (*P* = 0.005) and P0 (*P* = 0.01) and significant decreases in Satb2+ nuclei number at E15.5 (*P* = 0.002), E17.5 (*P* = 0.003) and P0 (*P* = 0.007). Counts were performed on three sections from two wild-type and two Dicer null cortices at each timepoint. Scale bars: 50 μm.

To assess the stage during development when the increase in numbers of Tbr1+ cells occurs, differentiation of Tbr1+ neurons and Satb2+ layer 2 to 4 neurons was monitored during cortical development from E13.5, before the onset of upper layer genesis, until birth (P0; Figure 3A). In both control and Dicer null cortices at E13.5 there was no significant difference in the number of Tbr1 positive neurons between the two genotypes (wt 13 ± 2.1, null 14 ± 2.4 neurons/100 μm width of cortex; Figure 3B). By E15.5, when wild-type cortical progenitor cells are primarily generating upper layer neurons, the Dicer null cortex contained slightly more Tbr1+ neurons (94 ± 6.8 neurons/100 μm width of cortex) compared to the wild-type (76 ± 4.6 neurons/100 μm width of cortex). In the wild-type cortex the numbers of Tbr1+ neurons reached a plateau of 76 ± 4.6 neurons/100 μm at E15.5 and remained constant until birth. However, in the Dicer null cortex the number of Tbr1+ neurons increased until birth, reaching 132 ± 4.7 neurons/100 μm at E17.7 and 208 ± 18.8 neurons/100 μm at birth/P0 (Figure 3B). By birth, the Dicer null cortex contained more than twice the numbers of Tbr1+ neurons than found in the wild-type cortex.

In both control and Dicer null cortex at E13.5 very few Satb2+ callosal projection neurons could be detected (wt 2 ± 1.3, null 2 ± 0.2 neurons/100 μm width of cortex; Figure 3C). Subsequently, wild-type cortex gained a significant number of Satb2+ upper layer neurons by E15.5 (100 ± 6 neurons/100 μm width of cortex), while very few Satb2+ cells were detected in the Dicer null cortex (9 ± 2 neurons/100 μm width of cortex). By E17.5, Satb2 positive neurons had begun to accumulate on the pial side of the Tbr1+, layer 6 neurons and could also be seen in the SVZ and interspersed with Tbr1+ neurons in layer 6 in the wild-type cortex (total number of Satb2+ neurons, 142 ± 0.2/100 μm width of cortex). In contrast, an average of 35 ± 8.9 Satb2+ neurons /100 μm width of cortex were found in the Dicer null cortex. By P0 almost all Satb2+ neurons had migrated and formed a distinct outer layer in the wild-type cortex (Figure 3A; 149 ± 9.0 neurons/100 μm width of cortex), whereas in the Dicer null cortex significantly fewer Satb2+ neurons were found (54 ± 6.8 neurons/100 μm width of cortex), and these were scattered throughout the cortical plate (Figure 3A).

### Progenitor cell populations in the Dicer null cortex

The large number of Tbr1+ neurons in the Dicer null cortex, and the accompanying absence of other projection neuron types, could be a consequence of several distinct processes. First, it could be due to premature, increased neurogenesis in the Dicer null cortex at an early stage of development, at the expense of the later production of upper layer neuronal types, which would be reflected in major changes in the numbers and proportions of apical and basal progenitor cells. Second, the multipotency of cortical stem and progenitor cells could be Dicer-dependent, resulting in their inability to generate the neuronal diversity normally found in the cerebral cortex. Third, the absence of Dicer could result in the selective cell death of upper layer, later born neurons.

**Figure 4:**
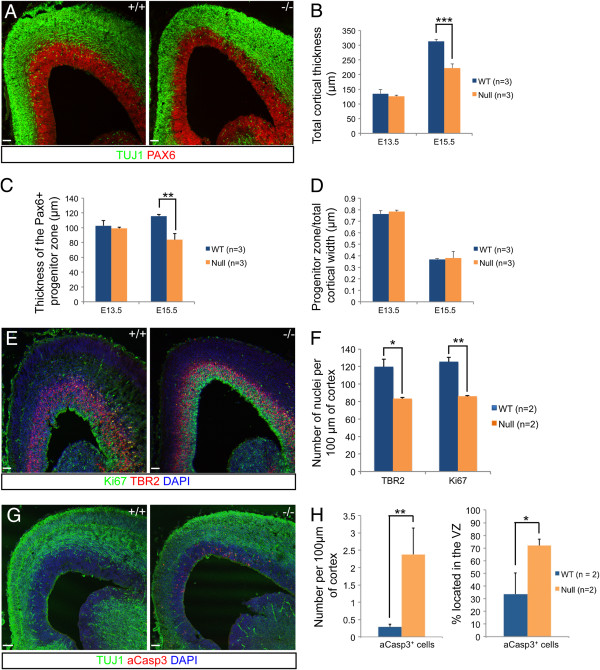
**Reduction in the number of cortical progenitor cells in the developing Dicer null cortex. (A-D)** The total cortical width and ventricular zone width were measured at three points across each section (medial, lateral and midpoint) and averaged. A total of three matched sections from two or three (E13.5 and E15.5, respectively) Dicer null and control littermate embryos were analysed. There was a significant reduction in both the total cortical width (*P* = 0.0006) and the ventricular zone (*P* = 0.003) by at E15.5 but not at E13.5. **(E, F)** There was a significant reduction (*P* = 0.02 and 0.001, respectively) in the number of basal progenitor cells (Tbr2+) and proliferating cells (Ki67+) in the E15.5 Dicer null cortex. **(G-H)** Cells expressing aCasp3 were far more prevalent in the Dicer null cortex at E15.5 compared to controls (*P* <0.01). A larger proportion of aCasp3+ cells were located in the VZ in Dicer null cortex than the wild-type (72.0% and 33.5%, respectively; *P* = 0.02). Counts were performed on three sections from two wild-type and two Dicer null cortices. Scale bars: 50 μm.

To assess whether the lack of upper layer neuron production was secondary to major changes in the cortical progenitor cell populations, the relative numbers of progenitor cells and neurons were quantified at E13 and E15 (Figure 4A-D). At E13, there was no difference in the size of the Pax6+ ventricular zone between control and Dicer null littermates. However, at E15 there was a significant, 20% reduction in the thickness of the Pax6+ VZ in the Dicer null cortex, which was reflected in the 20% decrease in Ki67+ cycling cells in the Dicer null cortex at this stage (Figure 4C). Furthermore, there was also a one-third reduction in Tbr2+ basal progenitor cells at E15 (Figure 4E, F). Together, this reduction in progenitor cell populations would result in an overall smaller cortex.

Measurements of the number of cells undergoing apoptosis at this stage, by immunostaining for cleaved Caspase-3 (aCasp3; Figure 4G, H) identified a significant, over five-fold increase in the total number of apoptotic cells in the Dicer null cortex, over 70% of which are in the VZ. This would indicate that the reduction in progenitor cells is due, in part, to increased cell death in this cell population.

### Dicer null progenitor cells generate Tbr1+ projection neurons late in development

The timecourse of appearance of Tbr1+ and Satb2+ neurons in the Dicer null cortex could be due to an early increase in neurogenesis, which would generate a transient increase in Tbr1+ deep layer neurons, deplete the progenitor pool and thus reduce upper layer neuron genesis later in development. For this to be compatible with the observation reported above of continual appearance of Tbr1+ neurons throughout cortical development, such an early increase in neurogenesis would have to be accompanied by a period of delayed terminal differentiation of the neurons generated in the first wave of neurogenesis. Alternatively, Dicer null progenitor cells could continue to generate deep layer, Tbr1+ neurons throughout the neurogenic period.

**Figure 5:**
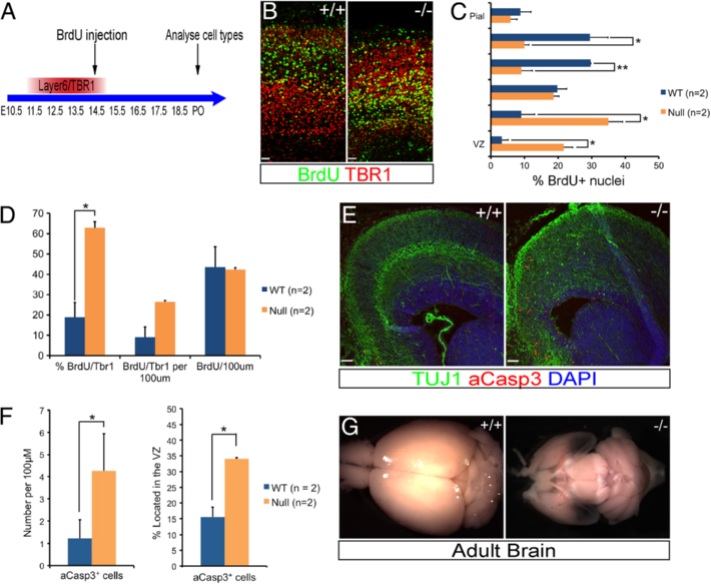
**Birthdating of Tbr1+ neuron genesis in the Dicer null cortex. (A)** Experimental design of neuronal birthdating experiment. BrdU was injected into timed-pregnant dams at E14.5, when the generation of Tbr1+ neurons is drawing to a close in wild-type embryos. BrdU incorporates into cycling progenitor cells and is subsequently maintained in newly born, postmitotic neurons. Tissue was collected at birth and coronal sections were analysed by immunofluorescence to determine the cell types generated over this period. **(B)** Immunofluorescence for BrdU and Tbr1 showed an increase in the number of double labelled cells in the Dicer null. **(C)** The cortex was divided into six regions of equal size and the BrdU + cells in each fraction were counted. In the Dicer null cortex a higher percentage of BrdU + cells were found in the bins located proximal to the VZ. **(D)** In the Dicer null cortex 63% of the cells labelled with BrdU were Tbr1+, compared with 19% in the wild-type (*P* = 0.03). There was no difference in neuronal output in the Dicer null cortex, as indicated by the total number of BrdU + cells per 100 μm. Counts were performed on three sections from two wild-type and two Dicer null brains. **(E)** Immunofluorescence at E17.5 showed an increase in apoptotic, aCasp3+ cells in both the VZ and the cortical plate. **(F)** Quantification of immunofluorescence images confirmed that, the Dicer null cortex contained a greater number of aCasp3+ cells per 100 μM of VZ (*P* = 0.04) and a higher proportion of these were located in the VZ (*P* = 0.01). **(G)** Gross anatomy of the brains of 2-month-old (adult) mice reveals a striking reduction in the size of the Dicer null cortex, which is almost completely absent with the ventral forebrain visible through the transparent cortical remnant. Scale bars 50 μm.

To distinguish between these possibilities, BrdU birthdating was carried out at E14.5, at the stage of cortical neurogenesis in the wild-type cortex at which the majority of cortical progenitor cells have switched from producing early born, deep layer neurons to generating upper layer neurons [30]. Birthdating analysis of the output of cortical progenitor cells at E14.5 in the wild-type and Dicer null cortex found that there was no significant difference in the number of BrdU positive neurons found in the P0 cortex (Figure 5). Therefore, there was no difference in neuronal output over this period in Dicer null cortex, compared to controls, and cell death is unlikely to be responsible for the change in numbers of upper layer neurons found in the Dicer null cortex. However, it is noteworthy that, whereas BrdU + neurons generated at E14.5 in the wild-type cortex are found mainly in the outer half of the cortex (Figure 5), the majority of BrdU + neurons in the Dicer null cortex are found in the deep layers. This is in line with previous reports of a defect in neuronal migration in the Dicer null cortex [17,19].

With respect to neuronal identity, as expected, only a small subset (19%) of the BrdU+ neurons were Tbr1+ neurons in the wild-type cortex. However, in the Dicer null cortex the majority of BrdU+ neurons were Tbr1+ neurons (65%; Figure 5D). Therefore, at a time when cortical progenitor cells normally have progressed from making deep layer neurons to making upper layer neurons, progenitor cells in the Dicer null cortex continue to generate deep layer neurons.

To assess the contribution of cell death to the observed phenotype, the numbers and radial position of aCasp3-positive cells in the Dicer null cortex were compared with those in wild-type littermates at E17.5 (Figure 5E, F). At E15.5, there was a significant increase in cell death in the Dicer null cortex and the majority of apoptotic cells in the Dicer null cortex were located in the VZ. At E17.5 a minority (<35%) of apoptotic cells were in the VZ, with the majority being apoptotic neurons in the cortical plate (Figure 5E, F). The progressive and generalised neurodegeneration in the Dicer null cortex is clear from the almost complete degeneration of the dorsal pallium observed in the P28 cortex (Figure 5G, H).

### Dicer null cortical progenitor cells maintain an early progenitor cell identity

**Figure 6:**
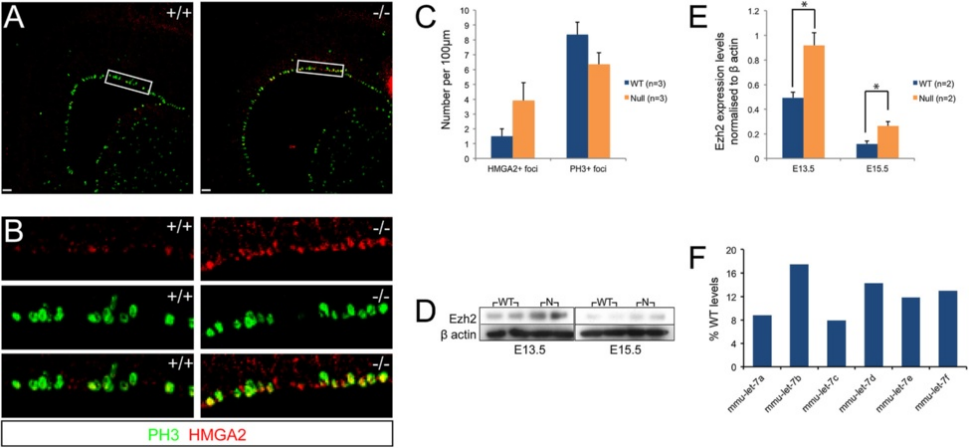
**Ezh2 and HMGA2 proteins are more highly expressed in Dicer null cortical progenitor cells during development.** Over the course of development neural progenitors become restricted to the generation of upper layer neuron types and this coincides with a decrease in Ezh2 and Hmga2 protein levels but the Dicer null cortex showed increased expression of both proteins compared to wild-type. **(A)** Hmga2+ foci were located along the ventricular surface in the wild-type and Dicer null E15.5 cortex confirming that Hmga2 is expressed in neural progenitor cells. **(B)** Magnification of boxed area in **(A)** showing overlap of Hmga2+ foci with the mitotic marker phospho-histone H3. **(C)** The number of Hmga2+ foci per 100 μm of ventricular zone was significantly increased in the Dicer null (*P* = 0.01) compared to the wild-type but despite the reduction in size of the ventricular zone, there was no difference in the number of mitotic cells. A total of three sections from three Dicer null and three wild-type littermate cortices were analysed. **(D)** Western blot of Ezh2 protein levels in the Dicer null cortex at E13.5 and E15.5 compared to the wild-type. Protein was extracted from two wild-type and two Dicer null brains at each time point. **(E)** Quantification of Ezh2 protein expression normalised to β-actin. **(F)** Expression of six let-7 family members in the E13.5 Dicer null cortex, compared with littermate controls. Expression is expressed as % of wild-type levels. Scale bars: 50 μm.

Hmga2 and Ezh2 are highly expressed in cortical progenitor cells early in the neurogenic period, and their progenitor cell expression becomes downregulated over the course of neurogenesis [27,31]. Deletion of Hmga2 reduces neural stem cell self-renewal during CNS development [31], whereas loss of Ezh2 function in the developing cortex results in reduced self-renewal and alterations in developmental timing [27]. Hmga2 protein was found expressed in apical cells of the ventricular zone at E15.5 (Figure 6) and was frequently, but not exclusively, co-expressed in cells expressing phospho-histone H3, indicating cells in late G2/M-phase. The number of Hmga2+ cells per 100 μm width of the VZ was significantly increased in the Dicer null cortex compared to wild-type (Figure 6). However, there was no difference in the number of mitotic (pH3+) cells between the wild-type and Dicer null cortex, indicating that the increase in number of Hmga2+ cells was not due to a general increase in proliferation or a gross alteration in cell cycle parameters (Figure 6).

Similarly, total amounts of the polycomb complex protein Ezh2 were significantly higher in the Dicer null cortex at both E13.5 and E15.5, compared with the wild-type cortex (Figure 6). Ezh2 was found expressed in Pax6+ cortical progenitor cells in both the wild-type and Dicer null cortex, as previously described [27], excluding the possibility that the absence of Dicer results in ectopic expression of Ezh2 in newly born neurons and, consequently, an increase in the total levels of Ezh2. Furthermore, there was no difference in the ratio between the thickness of the progenitor zone and total cortical thickness (Figure 6), indicating that the increase in Ezh2 levels in the Dicer null cortex is not due to changes in the cellular composition of the Dicer null cortex, but rather due to an increase in the amount of Ezh2 per progenitor cell.

Both Ezh2 and Hmga2 are known targets of the let-7 family of microRNAs [31-33]. Six let-7 family members are found in the set of the 40 most abundant miRNAs in the E13.5 cortex, and are reduced to between 8% and 16% of wild-type levels in the Dicer null cortex. The sustained high level of Ezh2 and Hmga2 proteins in the Dicer null cortex, and the marked reduction in let-7 miRNAs, is consistent with let-7 family members regulating translation of these two proteins in the developing cortex.

### Astrocytes are generated in the Dicer null cortex

**Figure 7:**
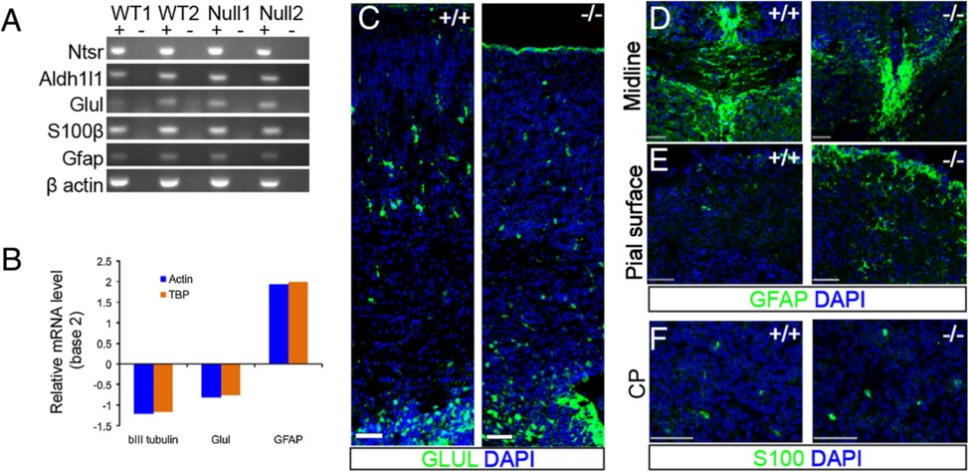
**Astrocytes are present in the neonatal Dicer null cortex. (A)** Astrocyte expressed mRNA transcripts; Ntsr2, Aldh1a2, Glul, S100β and Gfap and a β-actin control can be identified in whole cortex RNA extracts from two wild-type and two Dicer null pups by RT-PCR. cDNA generation was performed in the presence (+) and absence (-) of reverse transcriptase. **(B)** Quantitative RT-PCR for GFAP and Glul in the P0 wild-type and Dicer null cortex showed a reduction in βIII-tubulin and Glul mRNA. Conversely, GFAP mRNA levels were increased in the Dicer null cortex compared to wild-type controls. Average fold changes (expressed as base 2) are shown from *n* = 2 animals of each genotype are shown, compared against two control mRNAs, beta-actin and TBP. **(C-F)** Immunofluorescence for astrocyte markers in the wild-type and Dicer null neonatal (P0) cortex clearly identifies Glul + **(C)** and GFAP + astrocytes at the midline **(D)** and the pial surface **(E)**. S100+ **(F)** cells are also present throughout the cortical plate. Note the disorganised midline in the Dicer null **(C)**. Scale bars: 50 μm.

The continued production of early born, deep layer neurons in the Dicer null cortex, the near absence of upper layer neurons and the changes in progenitor cell gene and protein expression raise the possibility that changes in progenitor cell multipotency could also affect impact the switch from neurogenesis to gliogenesis. In wild-type cortical development, astrocytes are generated after E16 and accumulate in the cortex after birth. At birth (P0), RT-PCR for a set of genes expressed in astrocytes (GFAP, S100β, Glul (glutamine synthetase), Ntsr and Aldh1l1 [34], detected robust expression of the entire gene set in the Dicer null and wild-type cortex (Figure 7A), indicating that gliogenesis takes place in the absence of Dicer. Quantitative RT-PCR for GFAP and Glul confirmed that there was an increase in the amount of GFAP mRNA found in the Dicer null cortex, whereas the glial-expressed Glul and neuron-specific betaIII-tubulin were lower than in wild-type controls (Figure 7B).

**Figure 8:**
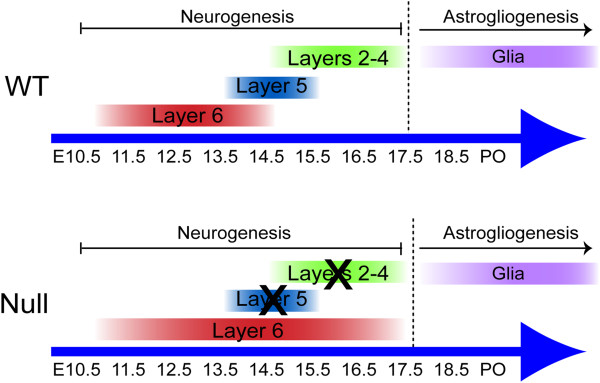
**Changes in cortical progenitor cell multipotency following loss of Dicer function.** The neurogenic period in mice begins at around E11 and ends at E17. Over this period all of the different classes of cortical projection neurons are generated in a fixed temporal order beginning with Tbr1+, layer 6 and subplate neurons. By E14.5 layer 6 neuron-genesis is waning and cortical progenitors are primarily producing CTIP2+ layer 5 cortical neurons. Finally, at around E15.5, upper layer genesis (characterised by expression of BRN2, CUX1 and Satb2) begins. Dicer null cortical progenitors fail to adhere to this stereotyped production of cortical projection neurons and generate Tbr1+ neurons throughout the neurogenesis period at the expense of other cell types. Despite this apparent lack of multipotency in the neuronal lineage Dicer null stem cells maintain the ability to undergo the higher order neurogliogenic switch to produce astrocytes.

The reciprocal changes in GFAP and betaIII-tubulin would suggest that there is a change in the relative proportion of astrocytes to neurons in the Dicer null cortex, although the reduced Glul levels are not consistent with this. As expected, cells expressing each of GFAP, S100 and Glul proteins were readily detected in the Dicer null cortex (Figure 7C-F), demonstrating that gliogenesis does take place in the Dicer null cortex. An increase in the density of GFAP-positive processes was observed, but was not consistent across the cortex and was more pronounced below the pial surface. This was not accompanied by an obvious difference in the number of Glul-positive cells. Therefore, although astrocytes are generated at the appropriate time in development, it is not clear that gliogenesis and glial differentiation is absolutely normal, compared with that in the wild-type cortex. However, in the presence of the reduced multipotency of cortical progenitor cells in the Dicer null cortex, as reflected in the failure of Dicer null progenitor cells to generate significant numbers of upper layer neurons, Dicer null progenitor cells maintain the ability to undergo the neuron-glial switch and generate astrocytes (Figure 8).

## Discussion

We report here a function for Dicer in regulating neural stem and progenitor cell multipotency and lineage progression in the developing cerebral cortex. We find that, in parallel with a progressive degeneration of postmitotic neurons, Dicer null cortical progenitor cells continue to generate deep layer neurons for significantly longer in the neurogenic period than in control cortices.

A number of previous studies have removed Dicer function in different parts of the developing nervous system and at different developmental stages [9]. There have been several different, and sometimes conflicting, phenotypes reported for Dicer loss of function in different parts of the CNS. For example, Dicer knockouts have been reported to result in increased astrogliogenesis in the cortex [19], but to repress the switch from neurogenesis to gliogenesis in the neural tube [20]. In the cortex, loss of Dicer function has been reported to have no effect on neurogenesis [17], to alter differentiation of early born neurons [16] and also to reduce the production of upper layer neurons [15]. Previous studies have shown that the timing of Dicer deletion contributes to these differences in phenotypes [16]. Given that deletion of Dicer does not result in an immediate loss of function, as the accumulated mature miRNAs are not synchronously removed from the progenitor cell population ([13] and this report), the progenitor cell phenotypes observed would suggest that the molecular mechanisms regulating multipotency and lineage progression are dosage sensitive. This would be consistent with our previous finding that cortical progenitor cells demonstrate Pax6 dosage-dependent phenotypes [35].

An emerging consensus from previous studies is that Dicer is required for neurogenesis, but not for neural stem cell self-renewal [13]. Increased apoptosis in the progenitor zones (VZ/SVZ) and in the cortical plate is commonly reported in the Dicer null CNS and has been interpreted as indicating that neural progenitor cells or newly born neurons undergo apoptosis in the absence of Dicer [15,16,18]. However, we have previously found that Dicer is critical for neural stem cell differentiation but not for self-renewal: reintroduction of Dicer is enough to restore the differentiation potential of the Dicer null neural stem cells [13]. One possibility is that the apoptotic cells seen in the proliferative zones in the Dicer null cortex are most likely cells that have exited the cell cycle and are attempting to undergo differentiation, but failing to do so and undergoing apoptosis. This would be consistent with the marked neuronal degeneration observed in the postnatal retina and cerebellum lacking Dicer function [21,22], and the almost complete progressive cortical degeneration that takes place in the early postnatal weeks reported here.

However, the severity of the postnatal neurodegeneration notwithstanding, cell death is not likely to account for the continued production of deep, layer 6 neurons observed in the Dicer null cortex throughout the neurogenic period. We confirm here that there is a general neurodegeneration in the Dicer null cortex that leads to almost complete degeneration of the dorsal pallium by adulthood, rather than a selective degeneration of upper layer cortical neurons. One possibility is that the phenotype of reduced cortical neuronal diversity reported here is due to a failure of terminal neuronal differentiation, rather than of cell fate specification at the level of cortical progenitor cells. In such a scenario, cortical progenitor cells successfully generate newly born neurons specified to differentiate into upper layer neurons, but the upper layer differentiation programme is critically dependent on Dicer and fails to execute. A corollary to this interpretation is that neurons that fail to differentiate to upper layer neurons default to a deep layer identity. This possibility cannot be completely excluded in the absence of a technical approach that removes both Dicer function and all microRNAs specifically in neurons, including those microRNAs inherited from its parent progenitor cell.

However, indirect evidence suggests that post-mitotic neurons are unlikely to be the locus of action of Dicer responsible for this phenotype. We do not observe cortical neurons with hybrid molecular identities, co-expressing transcription factors normally found exclusively in different projection neuron subtypes, for example Satb2, CTIP2 and Tbr1 [36], which might be expected if terminal differentiation was substantially aberrant. Furthermore, the neurons that are found in the Dicer null cortex fulfil all the criteria to be classified as layer 6/subplate projection neurons, both in terms of gene expression and their specific projections to the thalamus. Finally, the altered neuronal output found in the Dicer null cortex is accompanied by persistently high expression of two proteins, Ezh2 and Hmga2, which are normally expressed at high levels early in cortical development, reducing over time. High levels of both proteins are associated with regulation of neural stem cell self-renewal during development [27,31]. The persistent expression of both proteins at late stages of development suggests that Dicer null cortical stem/progenitor cells fail to change their molecular identity appropriately over time in the absence of Dicer and microRNAs, which reduces their competence to generate complex neuronal lineages. The marked reduction in let-7 miRNAs in the Dicer null cortex, which are known to regulate translation of both Ezh2 and Hmga2, also points to a role for miRNAs in regulating temporal changes in progenitor cell competence and multipotency.

Such a model for a role for Dicer in regulating the multipotency of neural stem cells in the developing cerebral cortex would be consistent with recent findings in the developing retina [18]. Loss of Dicer function in retinal progenitor cells leads to a reduced production of cells generated in the latter stages of the retinal neurogenic period. This is accompanied by a failure of retinal progenitor cells to alter their gene and protein expression over time in a manner typically observed in the developing wild-type retina. For example, Dicer null retinal progenitor cells fail to upregulate Sox9 expression after E16 [18]. In the cortical Dicer knockout, we observe more marked reduction in neuronal diversity, which we attribute to minor, but important, differences in the timing of Dicer deletion relative to the onset of neurogenesis in each system.

Mechanistically, by analogy with heterochronic mutations in *C. elegans,* it is possible that Dicer’s role in controlling multipotency, and thus developmental timing, in neural lineages is mediated via specific microRNAs. To date, no individual or groups of microRNAs have been found to control developmental timing in vertebrates. However, it is suggestive that let-7 family microRNAs are highly expressed in the developing cortex, are reduced in the Dicer null cortex, are known to target both Ezh2 and Hmga2 [31-33] and that both proteins show sustained increased expression in the Dicer null cortex. Further study is required to investigate the functions of individual microRNAs in regulating neuronal diversity and stem cell multipotency.

## Conclusions

Deletion of Dicer in cortical stem and progenitor cells before the onset of neurogenesis resulted in greatly increased production of the first-born cortical projection neuron class, Tbr1-expressing, deep layer and subplate neurons, with marked reduction in all later born cell types. Cortical neurons in the Dicer null cortex project primarily to the thalamus, with no detectable corticospinal projection, consistent with the presence of functional layer 6/subplate neurons and absence of layer 5. Birthdating analysis demonstrated that cortical stem/progenitor cells in the Dicer null cortex continued to generate Tbr1-expressing neurons late in development, at a stage when control stem/progenitor cells had progressed to generating upper layer projection neuron types. However, the switch to gliogenesis in the cerebral cortex was not grossly altered following Dicer deletion, despite the loss of multipotency and the failure of lineage progression. Therefore, loss of Dicer function does not lead to a complete developmental arrest in cortical stem and progenitor cells. Instead it would appear that Dicer is required for regulating cortical stem cell multipotency with respect to neuronal diversity, without affecting the larger scale switch from neurogenesis to gliogenesis.

## Competing interests

The authors declare they have no competing interests.

## Authors’ contributions

NS and TA participated in the design of the study, generated mice, carried out anatomical, gene/protein expression and molecular biology studies and wrote the manuscript, contributing equally. NV and ZM carried out the lipophilic dye tracing. FJL participated in the design of the study, carried out anatomical and protein expression studies and wrote the manuscript. All authors read and approved the final manuscript.

## Supplementary Material

Additional file 1 Microarray analysis of the most abundant microRNAs in control E13.5 cortex compared with Dicer-null cortex. Click here for file
